# Suitable Polymeric Coatings to Avoid Localized Surface Plasmon Resonance Hybridization in Printed Patterns of Photothermally Responsive Gold Nanoinks

**DOI:** 10.3390/molecules25112499

**Published:** 2020-05-27

**Authors:** Piersandro Pallavicini, Lorenzo De Vita, Francesca Merlin, Chiara Milanese, Mykola Borzenkov, Angelo Taglietti, Giuseppe Chirico

**Affiliations:** 1 Dipartimento di Chimica, Università di Pavia, viale Taramelli, 12–27100 Pavia, Italy; lorenzodevita01@universitadipavia.it (L.D.V.); francesca.merlin01@universitadipavia.it (F.M.); chiara.milanese@unipv.it (C.M.); angelo.taglietti@unipv.it (A.T.); 2 Dipartimento di Fisica “G.Occhialini”, Università Milano Bicocca, p.zza della Scienza, 3–20126 Milano, Italy; mykola.borzenkov@unimib.it (M.B.); giuseppe.chirico@mib.infn.it (G.C.)

**Keywords:** gold nanoparticles, photothermal effect, nanoink, inkjet printing, secure writing, anti-counterfeit

## Abstract

When using gold nanoparticle (AuNP) inks for writing photothermal readable secure information, it is of utmost importance to obtain a sharp and stable shape of the localized surface plasmon resonance (LSPR) absorption bands in the prints. The T increase at a given irradiation wavelength (ΔT_λ_) is the retrieved information when printed patterns are interrogated with a laser source. As ΔT_λ_ is proportional to the absorbance at the wavelength λ, any enlargement or change of the absorbance peak shape in a printed pattern would lead to wrong or unreliable reading. With the aim of preparing AuNP inks suitable for inkjet printing of patterns with stable and reliable photothermal reading, we prepared liquid solutions of spherical AuNP coated with a series of different polymers and with or without additional dispersant. The optical stability of the inks and of the printed patterns were checked by monitoring the shape changes of the sharp LSPR absorption band of AuNP in the visible (λ_max_ 519 nm) along weeks of ageing. AuNP coated with neutral polyethylenglycol thiols (HS-PEG) of mw 2000–20000 showed a strong tendency to rapidly agglomerate in the dry prints. The close contact between agglomerated AuNP resulted in the loss of the pristine shape of the LSPR band, that flattened and enlarged with the further appearance of a second maximum in the Near IR, due to plasmon hybridization. The tendency to agglomerate was found directly proportional to the PEG mw. Addition of the ethylcellulose (EC) dispersant to inks resulted in an even stronger and faster tendency to LSPR peak shape deformation in the prints due to EC hydrophobicity, that induced AuNP segregation and promoted agglomeration. The introduction of a charge on the AuNP coating revelead to be the correct way to avoid agglomeration and obtain printed patterns with a sharp LSPR absorption band, stable with ageing. While the use of a simple PEG thiol with a terminal negative charge, HS-PEGCOO(−) (mw 3000), was not sufficient, overcoating with the positively charged polyallylamine hydrochloride (PAH) and further overcoating with the negatively charged polystyrene sulfonate (PSS) yielded AuNP@HS-PEGCOO(−)/PAH(+) and AuNP@HS-PEGCOO(−)/PAH(+)/PSS(−), both giving stable prints. With these inks we have shown that it is possible to write photothermally readable secure information. In particular, the generation of reliable three-wavelength photothemal barcodes has been demonstrated.

## 1. Introduction

Nanoparticles (NPs) capable of relaxing thermally when irradiated at the wavelength of their absorption bands add interesting photothermal properties to the many peculiarities of matter at the nanoscale. NP of this kind are made of highly absorbing substances such as Prussian Blue [1], copper sulfide [2] or, more frequently, noble metals [3]. In the latter case, the absorption bands responsible of the photothermal effect are due to the well-known phenomenon of localized surface plasmon resonance (LSPR). Excitation at the wavelengths of such bands gives typically a weak fluorescent emission [4] unless pulsed lasers sources are used, generating in the latter case the more intense two photon luminescence (TPL) [5]. Continuous irradiation of LSPR bands with laser or wide spectrum continuous sources leads thus to a largely prevalent thermal relaxation [5]. The maximum of absorption of the LSPR bands (λ_max_) of Ag and AuNP is a function of their dimensions and shape, that are both tunable during synthesis [6]. In particular, by decreasing the symmetry of Ag and Au nanoparticles from that of a sphere to those of elongated, branched or more complex shapes, such as nanorods, nanostars, nanoplates and nanoshells, the absorption maximum can be easily shifted from the visible to the Near-IR (NIR) range [3,6] including the so-called ‘bio-transparent window’ (750–900 nm). Following this, many papers have been published proposing antitumoral [7] and antibacterial [8] photothermal through-tissues therapies by using such non-spherical NP. Besides giving a photothermal response in solution and in-vitro or in-vivo wet conditions, dry surfaces coated with patterns of photothermal NP display an even more intense effect [9,10]. With this in mind, we recently proposed a radically different application of the photothermal effect of NIR-absorbing gold nanostars (GNS), i.e., their use in a new approach in secure writing and anti-counterfeiting applications [11].

**Figure 1:**
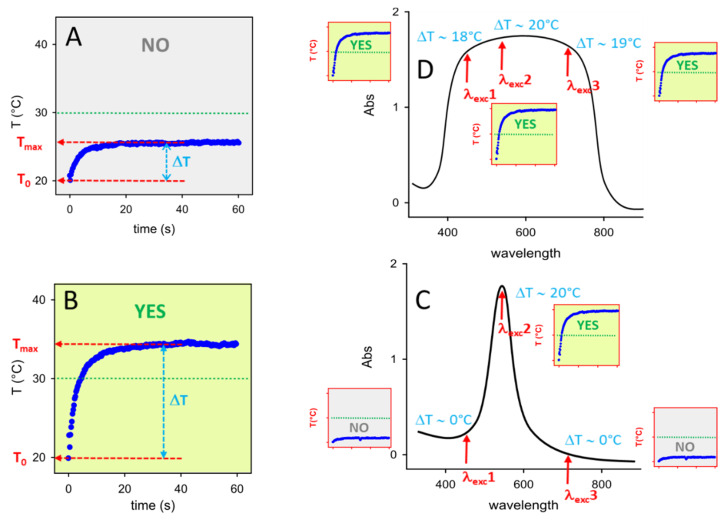
(**A**) and (**B**): examples of typical thermograms, i.e., T vs time profiles obtained irradiating a photothermally responsive printed pattern. ΔT values are calculated as the T differences T_max_ − T_0_ evidenced by the azure double arrows; T_0_ is 20 °C in both examples; a threshold ΔT of 10 °C has been chosen: in panel A ΔT < 10 °C (thermal answer: NO), in panel B ΔT > 10 °C (thermal answer YES). (**C**): visual sketch of how the photothermal answer changes by changing λ_exc_ with a print having a sharp absorption peak: small thermograms in the red-framed squares are what obtained (left to right) with λ_exc_1 (ΔT < 10 °C, answer: NO), λ_exc_2 (ΔT > 10 °C, answer: YES); λ_exc_3 (ΔT < 10 °C, answer: NO). (**D**): same, but with a large featureless absorpion band: at the same three λ_exc_ of panel C all the thermograms (red-framed squares) give ΔT > 10 °C, i.e., a YES answer.

Briefly, this approach includes: i) preparing photothermal NP inks, i.e., liquid solutions of NP suitable for inkjet printers; ii) printing patterns on a given bulk substrate (eg paper, glass); iii) interrogating the printed pattern by irradiating it with a laser source at a given wavelength (λ_exc_); iv) thermally reading the response with a thermocamera, obtaining a thermogram (T vs t, starting from T_0_ at t = 0) from which the maximum reached temperature (T_max_) is determined and the thermal answer is calculated as ΔT = T_max_ − T_0_, as sketched in Figure 1A,B.

A simple information is retrieved from this operation, as the ΔT answer may be YES or NO if it is higher or lower than a chosen threshold. In Figure 1, the chosen ΔT threshold is 10 °C, and the exemplificative thermograms of Figure 1A,B give a YES and NO answer, respectively. While we also proposed more sophisticated reading systems to create anticounterfeit secure printed patterns [11] like multiwavelength-interrogation photothermal barcodes, even the simple YES/NO thermal reading on a single irradiated point allows to obtain encoding/reading of secure information, as it requires complex technologies both to be written and to be read, and the correct keys are needed to interpretate the thermal response. The keypoint of the present paper is pictorially illustrated in Figure 1C,D. Given a sharp absorption band, the obtained ΔT is strongly dependent on λ_exc_ and once a threshold ΔT has been chosen (10 °C in the exemplifying sketch), a YES answer may become a NO answer even by relatively small changes in λ_exc_ (compare the response of λ_exc_2 with that of λ_exc_1 and λ_exc_3). In this case the knowledge of the correct λ_exc_ (and of the laser irradiance to be used) is one of the keys necessary to a user to verify if a printed point gives the exact expected answer. On the other hand, large absorption bands as in Figure 1D give the same thermal answer (YES, in the Scheme) for a large range of λ_exc_, eg for the same wavelengths of λ_exc_1, λ_exc_2 and λ_exc_3 of Figure 1C. With such large featureless absorptions the uniqueness of the correct wavelength of the laser source is lost, knocking down the security level of this key.

LSPR bands of AuNP are intense and sharp, and, in principle, such NP are ideal for preparing photothermally responsive inks suitable for this approach. However, as we have observed using GNS-containing inks [11], when AuNPs have been printed and the printed pattern is dry, the distance between nanoparticles may get so short that plasmon hybridization takes place [12,13]. This leads to large, featureless absorption bands, completely different from those observed in solution. Moroever, AuNPs are typically prepared in water as colloidal suspensions, while inks for inkjet printers must be liquid mixtures with higher viscosity and lower surface tension than water. Due to this, solvent mixtures have to be used (eg water with alcohols such as isopropanol and ethylene glycol) [11,14] and coatings must be grafted to AuNP to avoid agglomeration and precipitation due to solvent change. In summary, the correct AuNP coating must both grant stability in ink and avoid post-printing hybridization issues, i.e., it must be able to maintain AuNP at a sufficient inter-particle distance once a pattern is printed and dried out. At this regard, ionic polymers have been proposed as optimal coatings for gold nanostars [11] and nanorods [15]. In the present paper we use simple spherical gold nanoparticles (d = 17 nm), whose colloidal solutions have a sharp LSPR absorption in the visible (λ_max_ = 519 nm in water) and we examine a series of different coatings, including neutral or charged thiolated polyethylene glycol polymers (HS-PEG) of increasing length, both in the presence or absence of a typical thickening agent for traditional inks (ethyl cellulose, EC) [16] that may act as a dispersant. After this, we also examine the effect of alternate layers of oppositely charged ionic polymers. With all the coatings we have studied the NP stability in the ink, the sharpness vs flattening (due to hybridization) of the LSPR absorption in the printed patterns and the evolution with time of the LSPR absorption of both inks and prints. Ageing is of course an extremely relevant parameter for any real-life secure writing application. Finally, a proof of concept is presented of how to retrieve a correct or wrong photothermal answer at different λ_exc_ using AuNP inks prepared with optimal or unsuitable coatings.

## 2. Results and Discussion

### 2.1. AuNPs

**Figure 2:**
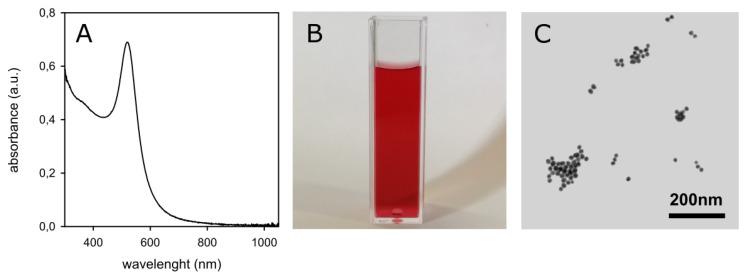
(**A**): absorption spectrum of a freshly prepared solution of citrate-coated AuNP. (**B**): photograph of the same solution. (**C**): TEM image obtained from the same solution.

The starting material for all inks preparations was citrate-coated spherical AuNPs prepared by the Turkevich method [17], i.e., by reduction of HAuCl_4_ with excess sodium citrate in bidistilled water (for details see Materials and Methods). Several 500 mL samples of such AuNP solutions were prepared in the course of the study. In all cases the expected sharp LSPR absorption band typical of small Au nanospheres was observed, with λ_max_ = 519 nm, see Figure 2A, imparting the typical intense purple-red color to the colloidal solutions (Figure 2B).

These aqueous colloidal solutions of citrate-coated AuNP are stable with time (no spectral changes in a 60 days range). Transmission electron microscope (TEM) imaging confirms the expected spheroidal shape of the prepared AuNP, Figure 2C, with average d = 17(±1) nm for all preparations. ζ-potential was −34(±2) mV (average of six preparations), due to the citrate coating. The Au(III) to Au(0) conversion yield can be safely considered ~100%, thanks to the noble nature of gold and to the large excess of the reductant (citrate anion). It has to be noted that following the Turkevich protocol, the total Au concentration in these colloidal solutions is 2.5 × 10^−4^ M (0.049 mg Au/mL). However, this value is too low for such solutions to be used as an ink component. As an example, we used colloidal solutions with 0.5–0.3 mg Au/mL (corresponding to 16.7–10 nM nanoparticles) for preparing photothermal inks with GNS [11]. Moreover, addition of alcohols is required to tune the viscosity to values suitable for inkjet printers. In this work we adopted a solvent mixture that we have already found to be optimal [9,11], i.e., 70% v/v aqueous AuNP solution, 20% v/v ethylene glycol and 10% v/v 2-propanol, with a viscosity and surface tension 1.92 cP and 40 mN/m, respectively, that is suitable for inkjet printers. Mixing the aqueous colloidal solution to the alcoholic components may induce agglomeration of citrate-coated AuNP. In addition, to prepare inks we needed 10-fold concentrated AuNP solutions (10 × solutions hereinafter), that can be obtained by ultracentrifugation, supernatant removal and pellet redissolution in 1/10 of the initial volume (see Materials and Methods for details). To make AuNP stable during all these procedures we coated them with PEG thiols, obtaining AuNP@HS-PEG.

### 2.2. AuNPs Coated with Neutral HS-PEG of Different Lengths

We used a series of thiolated PEG of general formula HS-(CH_2_CH_2_O)_n_-CH_3_, with molecular weights mw = 2000, 5000, 10000 and 20000 (n ~ 44, 112, 226 and 453, respectively). For sake of simplicity, we refer here to such polymers as HS-PEG_mw_. In addition, also the α,ω bifunctional polymer HS-(CH_2_CH_2_O)_n_-CH_2_COOH was used, with average mw 3000 (n ~ 66), referred to as HS-PEGCOOH in this paper. The –COOH group has typically a pKa of 4–5. Accordingly, in neutral water (pH 7) HS-PEGCOOH is deprotonated, bears a terminal negative charge, and can be referred to as HS-PEGCOO(−). Due to this, the properties of AuNP coated with HS-PEGCOOH are described in the Results and Discussion Section 2.4, that is dedicated to charged coatings, despite of the fact that HS-PEGCOOH has properties similar to those of all the neutral HS-PEG coatings.

**Figure 3:**
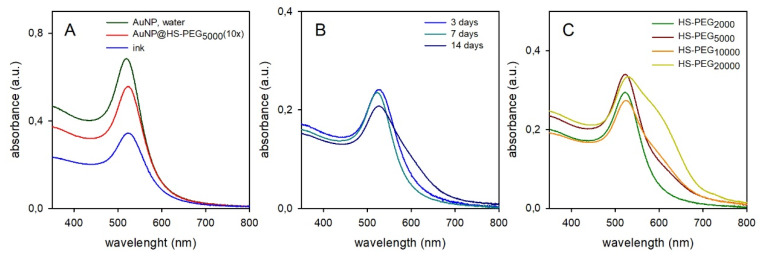
(**A**): absorption spectra of aqueous colloidal solutions of citrate coater AuNP (black) and 10 × AuNP@HS-PEG_5000_ (red) and of the ink obtained from the latter (blue). (**B**): evolution with time (3–14 days) of the absorption spectrum of an ink made with AuNP@HS-PEG_2000_ (time-color correspondances are shown in the palnel). (**C**): absorption spectra after 7 days for inks prepared with AuNP coated with the four different neutral HS-PEG (1 mm cell).

The coating step is carried out by adding the chosen HS-PEG in 2 × 10^−5^ M concentration to a volume of freshly prepared AuNP solution. HS-PEG concentration was chosen with this rationale: a spherical AuNP of 17 nm diameter has a mass of 4.97 × 10^−17^g; the Au(0) concentration in the citrate-coated AuNP solutions is 0.049 mg Au/mL; this leads to a 1.64 × 10^−9^ molar concentration of AuNP; an AuNP of 17 nm has ~ 6 × 10^3^ surface atoms [18], this meaning a concentration of potentially available surface Au atoms in the AuNP solution of ~ 9.8 × 10^−6^ M. Following also the obvious consideration that, due to steric crowding, not all the Au surface atoms can be coordinated by a thiolate group [19], we considered 2 × 10^−5^ M as a sufficiently large excess for HS-PEG to saturate the AuNP surface in the coating process. With all the used HS-PEG we observed a ~ 5 nm red-shift of the LSPR band maximum (λ_max_ = 524 nm) on grafting, due to the small local refractive index change when displacing citrate with ^−^S-PEG on the NP surface. As representative of all pegylations, the spectrum of AuNP@HS-PEG_5000_ (10 ×) is compared in Figure 3A (red line) with that of the starting AuNP solution (black line; identical spectra were obtained for all other HS-PEG). It has to be pointed out that the two spectra are recorded in 1 mm and 1 cm cells, respectively, and thus, in principle, their absorbances should be identical. However, pegylation and preparation of 10 × solutions requires repeated ultracentrifugation/redissolution cycles, that slightly decrease the AuNP quantity at each cycle, explaining the difference in Figure 3A. The spectrum of the ink obtained from the 10 × solution is also displayed in Figure 3A (blue line). Ink samples were obtained by adding 100 μL 2-propanol and 200 μL ethylene glycol to 700 μL of a 10 × pegylated AuNP aqueous solution.

Accordingly, in the just prepared ink the absorbance decrease is due to dilution with alcohols. However, no change in the spectrum shape is observed, indicating stability in the new solvent mixture at least on a short time stint (the spectrum was recorded 1 h after mixing). Also λ_max_ does not shift significantly, as expected from the small refractive index (n_D_) differences between water and the additives, (water n_D_ 1.33, 2-propanol n_D_ 1.37, ethylene glycol n_D_ 1.43), from the preponderance of water in the ink and from the small sensitivity to refractive index changes of the LSPR band of gold nanospheres (44 nm/RIU; RIU = refractive index units) [20]. On the other hand, inks show instability on ageing (weeks range), with the ink color changing from red to violet-blue (see SM1). This can be monitored spectroscopically. Figure 3B shows the representative case of AuNP coated with HS-PEG_2000_. While after 3 and 7 days the spectrum was still superimposable on the initial one, after 14 days a shoulder appeared at λ > 650 nm, as a typical indication of AuNP agglomeration [21]. Interestingly, inks prepared with AuNP coated with HS-PEG of increasing molecular weight showed that AuNP with longer PEG coatings undergo more significant AuNP agglomeration, when compared at the same ageing time (Figure 3C, 7 days). This counter-intuitive observation can be rationalized by observing the number of grafted HS-PEG per AuNP, Table 1 (data obtained from thermogravimetric analysis, SM2).

Such a number decreases on increasing the PEG length, most probably due both to hindering of the –SH function of the incoming polymers and to hindering of the available surface on the AuNP by the already grafted chains, two effects that become more significant with increasing the polymer dimensions. The slow agglomeration observed when AuNP coated with high mw PEG are dissolved in the alcohols/water ink mixture corresponds to the situation in which NP with low (i.e., partial) polymer coverage are dissolved in a poor solvent. In this case, we expect to find a minimum with negative free energy for approaching NP that interdigitate their polymer chains at NP-NP distances < 2L (L being the thickness of the coating polymer layer) [22].

Table 1 reports also the hydrodynamic radius and the ζ-potential values of the AuNP with different coatings. Citrate-coated AuNP have a highly negative ζ value (−34 mV) due to the citrate layer. Citrate is displaced when HS-PEGs are added. The latter adhere on the Au surface in the thiolate ^–^S-PEG form and this explains the observed negative ζ values, that however decrease with the increasing coating thickness (the ζ-potential is measured at the slipping plane), erasing any electrostatic contribution to AuNP stability.

**Figure 4:**
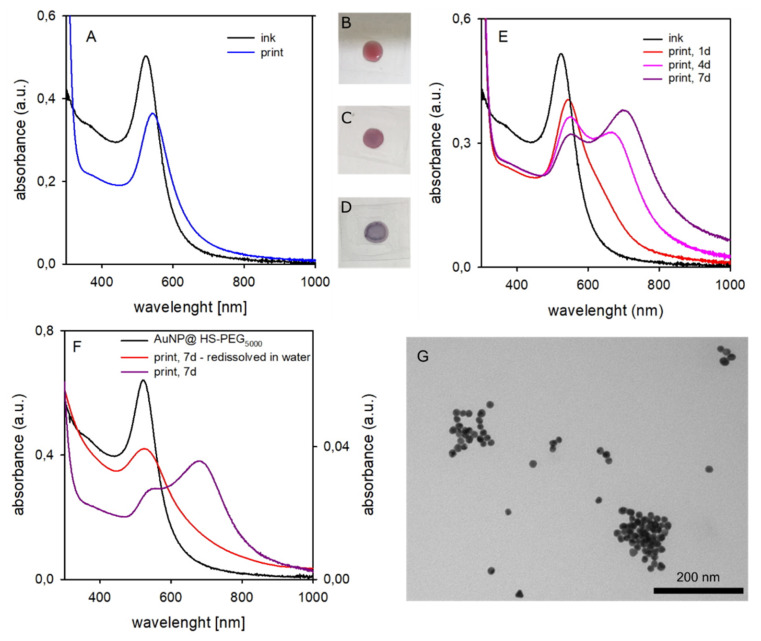
(**A**): absorption spectra of liquid ink containing AuNP@HS-PEG_5000_ (black) and printed on glass (dropcasted ink dried for 14 h, blue). (**B**): a drop of liquid ink just casted on glass. (**C**): same, after 14 h drying (corresponds to the blue spectrum in panel A). (**D**): same, after 7 days ageing (corresponds to the violet spectrum in panel E) (**E**): absorption spectra of the same ink (black) and evolution of the printed pattern after 1–7 days. (**F**): comparison of the absorption spectrum of the AuNP@HS-PEG_5000_ in water (black), of a 7 days aged print (violet), and after redissolution in water of the aged print (red). (**G**): TEM image of the AuNP redissolved from a 7 days aged print.

Using freshly prepared inks (ageing < 1 day), we printed patterns of ~ 1.0 cm^2^ on glass surfaces using the dropcasting protocol described in the Material and Methods Section 3.3.7. Such protocol allowed us to avoid the use of expensive research instruments for inkjet printing (not owned by our laboratories), while also allowing to prepare printed patterns mimicking the actually inkjet-printed ones. We used surface densities similar to those obtained with the optimal parameters standardized on a Dimatix Materials Printer DMP-2800 research inkjet printer (FUJIFILM Dimatix, Inc., Santa Clara (CA), USA) in previous collaborations [9,11]. Such parameters were 10 pL drops with 1681 drops mm^−2^ density and 1–11 printed layers with a 0.42 mg Au/mL ink concentration, corresponding to the 0.71–7.81 μg Au/cm^2^ range. In the present paper the inks concentration varied among 0.21 and 0.11 mg Au/mL, depending on the preparation. Using a 40 μL volume of inks, these spread over a ~1 cm^2^ surface (see Materials and Methods Section 3.3.7). This allowed us to print surfaces with a Au density between 8.4 μg/cm^2^ and 4.4 μg/cm^2^. We found a similar behaviour for all prints, almost independently on the mw of the HS-PEG coating (see SM3). Figure 4 shows the representative case of AuNP@HS-PEG_5000_. Printed surfaces were first examined after standard drying (14 h at 40 °C). A λ_max_ red-shift of 20 nm was observed in the absorption spectrum with respect to the liquid ink, blue line in Figure 4A. This is attributable to local refractive index changes, as after drying AuNP are no more dispersed in a water/alcohols mixture but surrounded by the PEG chains, that have an higher refractive index than water (eg n_D_ = 1.45 for PEG_200_ [23]. AuNP surface wetting by residual ethylene glycol (n_D_ = 1.43) from the ink formulation should be also taken into account, due to its higher boiling point (197.6 °C) with respect to water and 2-propanol (82.5 °C). A corresponding slight color change is perceivable also to the eye, when a just-dropcasted surface (a 40 μL drop of liquid ink on glass, Figure 4B) is compared with a 14 h-dried out surface, Figure 4C.

Following the evolution of the printed pattern with time by absorption spectroscopy (Figure 4E) revealed a progressive enlargement of the LSPR band with the formation and increase of a second maximum at longer wavelengths (λ_max_ = 700 nm after 7 days, dark violet spectrum). This is due to the agglomeration of AuNP, that leads to sufficiently short interparticle distances that LSPR hybridization takes place [13]. In agreement with spectral data, the color of 7 days aged printed patterns turns to blue-violet, Figure 4D. Printed patterns aged 7 days were redissolved in water by prolonged ultrasound treatment. Identically to the parent AuNP@HS-PEG_5000_ aqueous solutions (spectrum added for comparison in Figure 4F, black line), the obtained deep red solution displays an absorption spectrum with λ_max_ at 524 nm, Figure 4F, red line. However, the LSPR absorption is still significant at λ > 600 nm, suggesting a not complete separation into individual AuNP and the persistence of small agglomerates. This is consistent with TEM images obtained from solutions of redissolved AuNP@HS-PEG_5000_ printed patterns, Figure 4G, showing separate and agglomerated AuNP, still maintaining the original dimensions and shape, together with AuNP that have apparently started an authentic aggregation (i.e., fusion) process (see also SM4 for a larger image).

While the general behaviour of printed patterns is similar among all inks made with AuNP coated with neutral HS-PEGs, aggregation and consequent spectral changes parallel what observed for liquid inks, i.e., it is more significant for AuNP coated with the highest molecular weight polymers (see SM3). This clearly states that it is useless to increase the PEG dimensions in the coating to avoid that, in printed patterns, the dry AuNP could come sufficiently close one to the other to give plasmon hybridization. There is an apparent contraddiction with the hydrodynamic radius trend observed in Table 1 for aqueous colloidal solutions, as r_hyd_ increases with PEG mw. However, PEG chains have a good affinity for water, where they tend to outspread, while in the dry printed patterns we can hypothesize that their chains collapse on the AuNP surface, allowing close approach between AuNP.

### 2.3. Pegylated AuNP Codissolved with EthylCellulose (EC)

**Figure 5:**
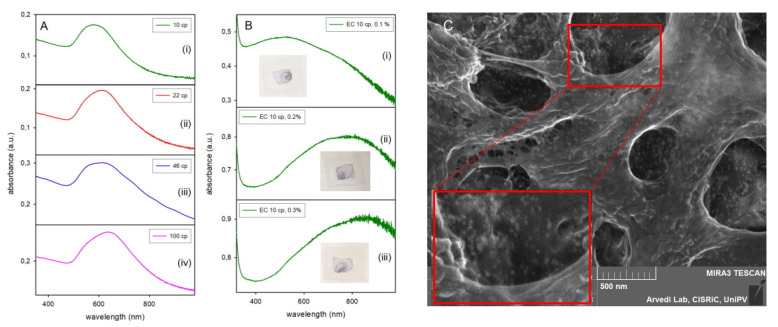
(**A**): absorption spectra of fresh prints (i.e., after 14 h at 40 °C) obtained with AuNP@HS-PEG_5000_ in ethanol with 0.1% EC of different viscosity (viscosity values are indicated in the panel). (**B**): absorption spectra of 7 days aged prints obtained with AuNP@HS-PEG_5000_ in ethanol with 10 cP EC at 0.1–0.3 % concentration. (**C**): SEM image taken on the print (ii) in panel (**B**) (EC 10 cP, 0.2%). The lower red-framed area is a 2 × enlargement of the upper red-framed area.

In the attempt of obtaining inks that fit the requirements of an inkjet printer while also assuring AuNP separation in the printed patterns, i.e., avoiding LSPR hybridization, we added EC, a non- volatile polymer, to the AuNP liquid inks. The aim was to keep AuNP dispersed in the dry prints, i.e., statistically separated and mechanically immobilized within the dispersant matrix. EC was chosen as it is used as standard dispersant and binder in the formulation of nanoparticle-based inks for inkjet printers [16]. EC scarce solubility in water and aqueous mixtures forced us to use ethanol as the solvent. AuNP@HS-PEG_5000_ were dissolved in ethanol, in which they were stable at least for 24 h, showing a sharp LSPR absorption slightly red shifted with respect to water (λ_max_ 528 nm) due to the refraction index change (see SM5). Addition of 0.1% w/v EC was carried out on 1.5 mL ethanolic AuNP solution, using commercial EC with η (viscosity) of 10, 22, 46 and 100 cp (for all the ECs (ethyl cellulose) samples the nominal viscosity η reported by the seller (Sigma Aldrich, Milano, Italy) in the specification sheets refers to 5% EC solutions in 80:20 toluen/ethanol). The nominal degree of methoxy substitution on the D-glucose units of cellulose is 48% for all the used products, so the differences in η are all attributable to differences in mw, that may be empirically calculated as 243000, 378000, 571000 and 882000 for EC with 10, 22, 46 and 100 cP viscosity, respectively (mw = k(η)^n^, where k and n are empirical constants that depends on the method used to determine mw; mw mentioned in this paper are calculated using typical values reported for EC, k = 66.96 × 10^3^, n = 0.56 [24]). In all cases, no significant LSPR shift was observed on EC addition. Ink stability was checked by absorption spectroscopy, observing that after 4 days only the solution with 10 cP EC still showed an acceptable spectrum (comparable to that of the PEG-coated AuNP dissolved in pure ethanol), while solutions with ECs with higher η presented a shoulder at ~ 700 nm, already indicating aggregation (SM6). Nevertheless, glass surfaces were printed using freshly prepared inks with all the four EC additives. A large, shifted absorption band was always observed after the usual 14 h drying process, Figure 5A. We hypothesise that this is due to aggregates of AuNP with different overall shape and dimensions. Further studies were carried out using the EC with lower viscosity (10cp), that was empirically choosen due to the closer similarity of the absorption spectrum of its printed patterns (Figure 5A (i), λ_max_ = 580 nm) with the original AuNP LSPR band. Ethanolic inks containing EC 10cP in different w/v percentages (0.05–0.3%) were prepared and glass surfaces printed. Absorption spectra after an ageing time of 7 days are shown in Figure 5B for 0.1, 0.2 and 0.3% solutions.

A few observations can be made here. First, the use of pure ethanol with added EC caused the spreading of the dropped ink on a larger area (actually all the area delimitated by a PDMS fence, see Materials and Methods) and resulted in a strong coffee stain effect, as it can be seen by the photographs of the printed slides (Figure 5B, insets). Prints changed to a blue color, as expected from the large range of absorption that includes visible and NIR, and a more significant λ_max_ red shift was observed in the spectra for higher EC concentrations. These results discouraged the use of hydrophobic polymeric additives in inks, at least as long as AuNP are coated with hydrophilic polymers. Second, AuNP dramatically agglomerate in the liquid ink and in the dried out printed patterns, with the complete loss of the original sharp LSPR band shape. The longer was the EC polymer chain and the higher was the additive concentration, the more intense was this phenomenon. Although we have discarded and not further investigated these systems, we can hypothesize that the presence of the non-volatile hydrophobic polymer in the printed patterns promotes segregation of the hydrophilic AuNP during the drying process, with the formation of clusters of nanoparticles of different shapes and dimensions. This hypothesis is consistent with the increasing degree of LSPR red shift with increasing EC concentration. SEM images on printed patterns allow to visualize the EC matrix but also the AuNP, see Figure 5C (0.2% EC printed sample). AuNP can be spotted both as isolated particles and as aligned or cropped groups (see in particular the enlarged section framed in red). The different type and degree of aggregation is consistent with the large, featureless absorption band, that is generated by the superimposition of different LSPR hybridizations.

### 2.4. Coating with Ionic Polymers

**Figure 6:**
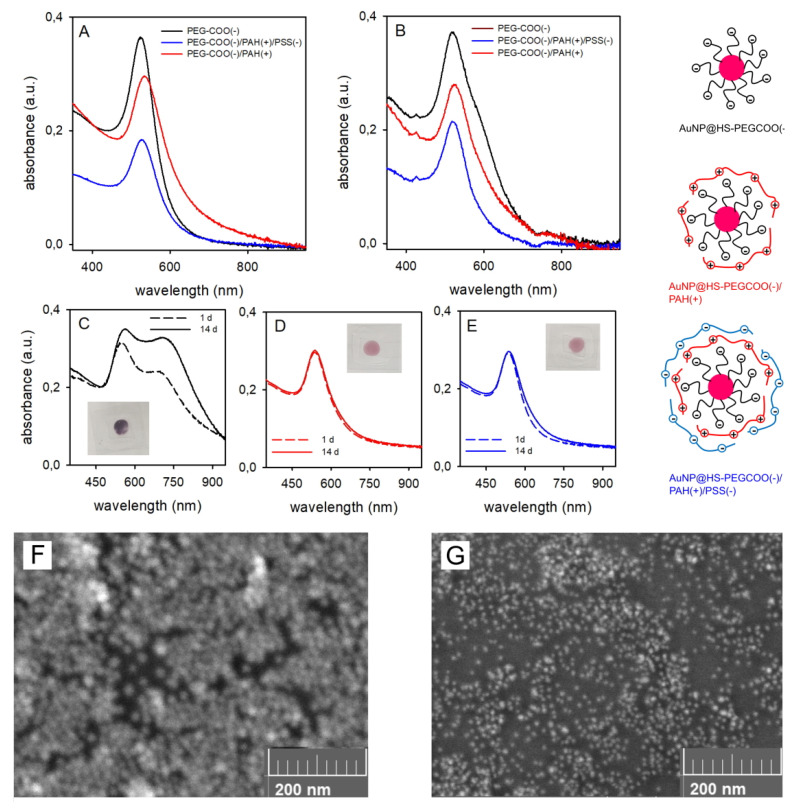
(**A**): absorption spectra of as-prepared inks; (**B**): inks after 7 days ageing; (**C–E**) absorption spectra of dry printed patterns after 1 day (dashed lines) and 14 days ageing (solid lines) for AuNP@HS-PEGCOO(−) (**C**), AuNP@HS-PEGCOO(−)/PAH(+) (**D**), AuNP@HS-PEGCOO(−)/PAH(+)/PSS(−) (**E**); insets are photos taken on 14 days aged prints; F-G: SEM imaged of 14 days aged prints with AuNP@HS-PEGCOO(−) (**F**) and AuNP@HS-PEGCOO(−)/PAH(+)/PSS(−) (**G**).

The next step of this study was to coat AuNP with ionic, charged polymers. A first HS-PEGCOOH (mw 3000) layer was grafted on the surface as already described for the neutral HS-PEG, in order to have both sterical stabilisation and a significantly negative ζ-potential for the AuNP. We measured ζ = −22 mV in neutral water. The negative charge is due to the carboxylic acid moieties, that are fully deprotonated at pH 7, as the typical pKa of a –COOH groups is in the 4–5 range (eg the pKa of acetic acid is 4.76). Accordingly, we describe AuNP coated with this polymer as AuNP@HS-PEGCOO(−), see sketch in Figure 6. The ζ value is consistent with those reported in Table 1 for neutral HS-PEG coatings with mw 2000 and 5000 (−13 and −6 mV, respectively) as in the latter cases the weakly negative values are due to the residual influence at the slipping plane of the remote negative thiolate groups grafted on the AuNP surface. From TGA analysis (SM2E-F) we calculated the number of polymers per AuNP as 1532, slightly lower than what found for the longer but neutral HS-PEG_5000_. This is coherent with what we observed with HS-PEGCOOH and GNS [25] and is attributable to the electrostatic repulsive effect between grafted and incoming polymers. Overcoating with PAH, a positively charged ionic polymer, was obtained by electrostatic adhesion of the polymer chains to the negatively charged AuNP@HS-PEGCOO(−). PAH was added in 2 x 10^−5^ M concentration to AuNP@HS-PEGCOO(−) solutions, as this quantity demonstrated sufficient to obtain the maximum PAH coating in the AuNP@HS-PEGCOO(−)/PAH(+) complexes (see Figure 6 for a sketch). These have a ζ-potential of +36(2) mV after 2 h equilibration at room temperature and two cycles of ultracentrifugation-redissolution in bidistilled water to remove non adhering PAH (final pH in the 5–6 range). The use of larger concentrations of PAH did not lead to an higher ζ-potential, suggesting complete coating with the chosen concentration. Finally, these complexes were also further overcoated with the negatively charged polymer PSS using an identical synthetic procedure to obtain the AuNP@HS-PEGCOO(−)/PAH(+)/PSS(−) complex (sketch in Figure 6). This displayed a negative ζ-potential of −27(2) mV (final pH ~ 6). It has to be pointed out that in the slightly acidic pH range of these solutions and up to strongly basic pH values (eg 9 or higher), the ζ-potential values do not change, as all the polymers maintain their ionic state and their typical charge. First, as we have already discussed, a –COOH function is prevalently deprotonated at pH > 5. Then, PAH is a polyamino polymer containing only –NH_2_ functions, and protonated primary amines have typically pKa values > 10 (eg pKa = 10.60 for *n*-butylammmonium [26]. Finally, PSS has –SO_3_^−^ groups that are deprotonated up to pH 14 (eg, benzenesulfonic acid is a strong acid [27]). The absorption spectra in water show a sharp LSPR maximum for all complexes, with negligible λ_max_ variations (< 5 nm) with any of the coatings. TGA and DLS measurements (Table 2) gave consistent results, showing an increasing quantity of coating material both as an absolute value and in the ratio with respect to the Au content, and an increase of the hydrodynamic radius on stepping from AuNP@HS-PEGCOO(−) to AuNP@HS-PEGCOO(−)/PAH(+) and to AuNP@HS-PEGCOO(−)/PAH(+)/PSS(−). Ink preparation was carried out using again the standard formulation of 70%(v/v) aqueous component (containing the coated AuNP), 20% v/v ethylene glycol and 10% v/v 2-propanol. Prior to mixing with alcohols, 10 × aqueous solutions of coated AuNP were analyzed by ICP-OES to determine Au concentration (data listed in Table 2). Inks were prepared either with the pure colloidal solution (AuNP@HS-PEGCOO(−)/PAH(+) case) or with the colloidal solutions diluted with a calculated volume of bidistilled water (AuNP@HS-PEGCOO(−) and AuNP@HS-PEGCOO(−)/PAH(+)/PSS(−) cases) so to have all inks with the same Au concentration of 0.11 mg/mL.

The lower Au concentration in the 10× aqueous solution of AuNP@HS-PEGCOO(−)/PAH(+) is due to the repeated ultracentrifugation cycles required to remove the excess PAH and to the tendency of PAH-coated NP to adhere to the plastic walls of the test tubes, resulting in a less efficient redissolution [25]. Table 2 also reports λ_max_ values of inks absorption spectra recorded 1 h after preparation. Small λ_max_ variations were observed with respect to water, but a sharp LSPR band of identical shape as in water (Figure 6A) was obtained in all cases. However, for the ink containing AuNP@HS-PEGCOO(−), similarly to what described for all the PEG-coated AuNP, ink ageing (7 days) resulted in the formation of a shoulder at longer wavelength, Figure 6B. On the contrary, the spectra of inks with AuNP overcoated with PAH and PSS did not change in 7d (Figure 6B), indicating ink stability. Printing was carried out with the usual procedure using freshly prepared inks, and the stability of the printed patterns was monitored again by absorption spectroscopy. Figure 6C–E compares the spectra recorded 1 day and 14 days after printing for the three inks. While prints with the AuNP@HS-PEGCOO(−) ink showed the growth of a band at longer wavelength (λ_max_ 720 nm), due to AuNP agglomeration, both inks with AuNP overcoated with ionic polymers displayed an excellent peak shape constancy. The insets of Figure 6C–E display the visual aspect of prints after 14 days. Prints with the AuNP@HS-PEGCOO(−) ink turned into a blue-violet color, while the original red-purple color was maintained for prints with the inks containing AuNP further coated with PAH and PSS. To further consolidate this observation, Figure 6F–G display SEM images taken on printed glasses (14 days ageing). A sharp difference was observed between an AuNP@HS-PEGCOO(−) print, Figure 6F, and an AuNP@HS-PEGCOO(−)/PAH(+)/PSS(−) print, Figure 6G. In the former, AuNP are thoroughly aggregated, while in the latter they are all sharply separated. A SEM image for a 14 days aged print from a AuNP@HS-PEGCOO(−)/PAH(+) ink, included in the Supplementary Materials (SM7), showed analogous features as those obtained with the AuNP@HS-PEGCOO(−)/PAH(+)/PSS(−) ink).

Noticeably, also in the case of the AuNP@HS-PEGCOO(−) prints, redissolution in water reverted agglomeration, yielding well separated AuNP (TEM image in SM8) with an absorbance identical to that recorded before ink formation (SM8).

### 2.5. Photothermal Reading of Secure Information

**Figure 7:**
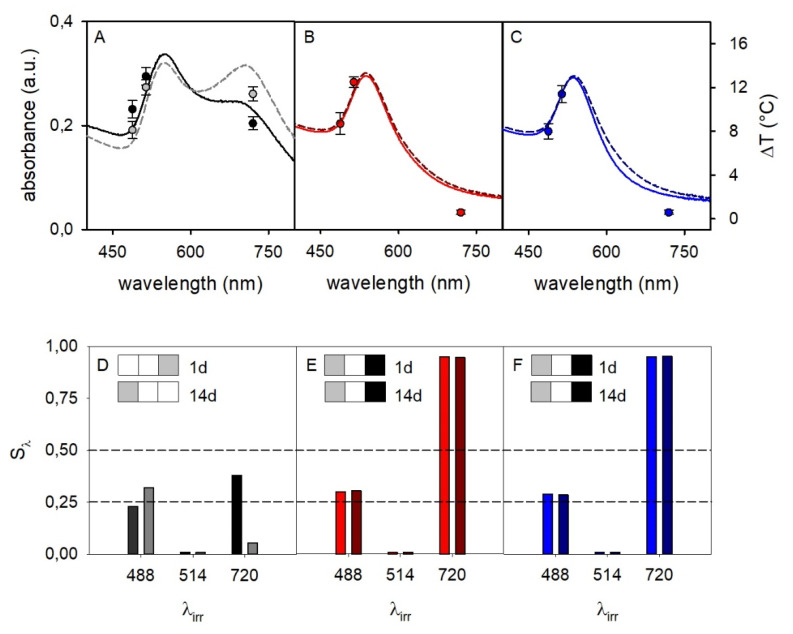
(**A–C**): absorption spectra (left vertical axis; full lines 1 day after print, dashed lines 14 days after print) and ΔT_λ_ values (circles, right vertical axis) for prints on glass slides with inks containing AuNP@HS-PEGCOO(−), AuNP@HS-PEGCOO(−)/PAH(+) and AuNP@HS-PEGCOO(−)/PAH(+)/PSS(−), respectively; panel A displays ΔT_λ_ values both for 1 day after print (black circles) and for 14 days after print (grey circles), while in panel (**B**) and (**C**) the ΔT_λ_ values at 14 days are not displayed as they are superimposable to those at 1 day. (**D–F**): S_λ_ values and relative three-wavelength barcordes for the same sequence of prints, after 1 day (left bars at a given wavelength) and after 14 days (right bars).

Once we obtained inks that give prints with the desired spectral stability, we carried out a proof-of-concept study on writing secure photothermal information. We used both AuNP@HS-PEGCOO(−)/PAH(+) and AuNP@HS-PEGCOO(−)/PAH(+)/PSS(−) inks, and compared the results with a print of the AuNP@HS-PEGCOO(−) ink. The photothermal response was recorded with interrogation of printed patterns with 1 day and 14 days ageing. The photothermal studies were carried out by irradiating printed patterns with laser sources at three different wavelengths (λ_irr_), 488, 514 and 720 nm. This is the set of sources that falls inside the absorption range of the prints and that is currently available in our laboratories. We followed a protocol that we have already successfully adopted [10,11]. Briefly, using a E40 thermocamera (FLIR System, Inc., Santa Barbara, CA, USA)) we read a 320 × 240 pixels thermal image, inside which we define a ROI (region of interest) that includes the laser-irradiated area. We run data analysis determining the maximum temperature inside the ROI (± 0.1 °C accuracy). At a λ_irr_ at which the prints display a significant LSPR absorption, a typical steep ascending T vs time profile is observed on irradiation, turning into a plateau in less than 10 s (thermograms sketched in Figure 1 give qualitatively similar examples; SM9 reports actual thermograms for this study). From such data we obtain ΔT_λ_ = T_max_ − T_0_, with T_max_ = temperature of the plateau at a given λ of irradiation and T_0_ = temperature before irradiation. The ΔT_λ_ data for prints with the three inks are displayed in Figure 7, superimposed to the absorption spectra of the prints.

The expected ΔT_λ_ vs λ_irr_ trend was observed, approximately following the absorbance spectra profile. Noticeably, comparison of spectra at 1 day and 14 days after printing (solid and dashed lines, respectively) evidenced a perfect stability of the prints with inks of AuNP overcoated with PAH and PAH/PSS, while a very significant further spectral change took place for AuNP@HS-PEGCOO(−) ink prints. Among the three inks prints, the most significant differences were observed when irradiating at 720 nm. The absorbance at 720 nm was high for prints with AuNP@HS-PEGCOO(−) inks after 1 day, due to aggregation and plasmon hybridization, and evolved to an even higher absorbance after 14 days. As a consequence ΔT_720_ = 8.4 °C (1d) and 11.4 °C (14 days), Figure 7A. On the other hand, in the prints obtained with inks of AuNP@HS-PEGCOO(−)/PAH(+) and AuNP@HS-PEGCOO(−)/PAH(+)/PSS(−) the coatings prevented aggregation and plasmon hybridation, keeping the original shape of the band unchanged after both 1 day and 14 days. Accordingly Abs_720_ is negligible and ΔT_720_ too (< 1 °C). As an example of application of the YES/NO reading scheme highlighted in the Introduction [11], if ΔT = 5 °C was chosen as the threshold for a YES answer with λ_irr_ = 720 nm, the prints with AuNP@HS-PEGCOO(−)/PAH(+) and AuNP@HS-PEGCOO(−)/PAH(+)/PSS(−) inks would give a NO answer, that is the correct one for an unaltered print. On the other hand, prints with the AuNP@HS-PEGCOO(−) ink, would give a YES answer, that is wrong (altered print). Beside the application of this simple concept, a robust way to obtain a more complex information from photothermal data has been introduced in our previous papers [11], that eliminates possible uncertainties due to laser power oscillations or ink concentration. A multiwavelength test with multiple levels can be set by defining a normalized temperature signature:

S_λ_ = (ΔT_λ__,max_ − ΔT_λ_)/ ΔT_λ__,max_

where ΔT_λ__,max_ is the largest T increase among those obtained with the available set of laser sources (ΔT_514_ in our case) and ΔT_λ_ is the T increase obtained when irradiating at any wavelength λ. A three level answer generating a three-color barcode can be established by choosing such levels as:

S_λ_ ≤ 0.25    barcode color: white

0.25 < S_λ_ ≤ 0.5  barcode color: grey

S_λ_ > 0.5    barcode color: black

In this study we have only three available laser sources, so we can generate a barcode with three bars. However, these are enough to establish a clear difference between a correctly printed surface, like those with AuNP@HS-PEGCOO(−)/PAH(+) and AuNP@HS-PEGCOO(−)/PAH(+)/PSS(−) inks, and a print with unsufficiently protected AuNP i.e., that with AuNP@HS-PEGCOO(−) ink.

Figure 7D–F show the S_λ_ values for the three prints and the corresponding three-wavelengths barcodes. Identical barcodes are obtained for the PAH- and PAH/PSS-overcoated AuNP inks (Figure 7E,F), that do not change after 14 days. On the contrary, the print with the AuNP@HS-PEGCOO(−) ink gives a different barcode at 1 day that, in addition, changes with time (14 days). It has to be noted that this result has been obtained by choosing a given surface concentration of gold, the three irradiation wavelengths and the three S_λ_ levels. Even considering a very simple printed pattern as in this work, all these parameters are the keys in which information can be hidden, i.e., their values are all needed both to write and to read the correct multiwavelength barcode. Of course, a stable ink that produces a print with a sharply peaked and time stable absorption is necessary. We have here shown that inks made of AuNP@HS-PEGCOO(−)/PAH(+) and AuNP@HS-PEGCOO(−)/PAH(+)/PSS(−) are suitable for this purpose. Finally, it has to be stressed that periodical check along a 2 months period of the inks and of the prints obtained with the PAH and PAH/PSS coated AuNP gave unmodified spectra.

## 3. Materials and Methods

### 3.1. Materials

Tetrachloroauric acid trihydrate (99.99%), sodium citrate dihydrate (≥ 99%), Triton X-100 (laboratory grade), sodium borohydride (≥ 98%), silver nitrate (> 99%), poly(ethylene glycol) methyl ether thiol (mw 2000), poly(allylamine) hydrochloride (PAH, mw 50000), sodium poly(4-styrene sulfonate) (PSS, mw 70000), nitric acid (≥ 65%), nitric acid (1M), hydrochloric acid (≥ 37%), sulfuric acid (95.0–97.0%), hydrogen peroxyde (30% w/w), ethanol (≥ 99.8%), 2-propanol (99.5%), and ethyl cellulose (10 cP; 22 cP; 46 cP; 100 cP) were bought from Sigma Aldrich (Milano, Italy); α-mercapto,ω-carboxy poly(ethylene glycol) (mw 3000), poly(ethylene glycol) methyl ether thiol (mw 5000), poly(ethylene glycol) methyl ether thiol (mw 10000), and poly(ethylene glycol) methyl ether thiol (mw 20000) were bought RAPP Polymere (Tübingen, Germany); Ethylene glycol (99.5%) and ammonia solution in water (30% w/w) were bought from Carlo Erba Reagenti S.p.A. (Milano, Italy) Glass coverslides (22 × 26 mm, 0.14 mm thickness) were bought from Delchimica Scientific Gòassware (Napoli, Italy).

### 3.2. Instrumentation

We used a Sonorex sonicating bath (Bandelin electronic, Berlin, Germany). Ultracentrifugation was carried out with a Z366 ultracentrifuge (Hermle Italia, Rodano, Italy). UV-Vis-NIR absorption spectra were recorded on Cary 50 and Cary 6000 spectrophotometers (Varian, Agilent, Santa Clara (CA), USA); spectra on solutions were taken in 1 cm or 1 mm glass or quartz cuvettes, spectra on glass slides were taken using a dedicated Varian sample holder). Measurement of pH was carried out with a pH-meter (pH 50 model, XS Instruments, Carpi, Italy) with an Orion 91022 BNWP combined glass electrode (Thermo Scientific, Monza, Italy) that was calibrated before measurements with solutions buffered at pH = 4 and pH = 7. Dynamic Light Scattering (nanoparticles dimensions) was performed with a Zetasizer Nano ZS90 instrument (Malvern Panalyticals, Malvern, UK); the same instrument was used for ζ-potential measurements, by using a dedicated dipcell electrode (1 mL samples of colloidal solutions for both techniques). ICP-OES analysis were carried out on an Optima 3000 DW system (Perkin Elmer Italia, Milano, Italy). SEM images were acquired with a Mira XMU series field emission scanning microscope (FEG-SEM) (Tescan, Brno, Czech Republic) at the Arvedi Centre (University of Pavia). Thermogravimetric analyses were carried out on Q5000 instrument (TA Instruments, New Castle (DE), USA). TEM images were taken on JEM-1200 EX II 140 instrument (Jeol Italia SPA, Basiglio, Italy) at the University of Pavia Centro Grandi Strumenti (CGS), on parlodion-coated copper grids, after drying a dropping of 10 microliters of solution. Thermograms were recorded with a FLIR E40 thermocamera driven by the Flir Tools + dedicated software.

### 3.3. Methods

#### 3.3.1. Glassware and Glass Coverslides Cleaning

Before any synthesis involving nanoparticles, glassware underwent a purification procedure to remove any possible traces of metal ions [28], i.e., it was rinsed with bidistilled water and then cleaned by filling it with aqua regia (3:1 v/v HCl 37% and HNO 3 65%) for 20 min, after which time the oxidant mixture was removed and the glassware filled with bidistilled water and sonicated for 3 min. The last water washing procedure was then repeated 2 further times. Finally glassware was kept at 120 °C in an oven for 2 h. Coverslides were washed with the same procedure, using staining jars filled with aqua regia, in which six or eight coverslides were kept in a vertical position.

#### 3.3.2. AuNP Synthesis

Spherical AuNP were prepared with the standard Turkevich method [17]. Briefly, 87 μL of a 1.44 M tetrachloroauric acid solution in water were added to 500 ml of boiling bidistilled water. Heating was then switched off and under 25 mL of a 1.7∙10^−2^ M sodium citrate dihydrate solution in water were added under magnetic stirring. The synthesis was considered complete after further 2h of stirring. Total Au concentration in the final solution is 2.4 × 10^−4^ M.

#### 3.3.3. AuNP Coating with HS-PEGs

Pegilation (surface coating with HS-PEGs) was obtained by treating at room temperature a given volume of the citrate-coated AgNP stock colloidal solution with a quantity of the chosen HS-PEG (added as a solid) so to reach a 2.0 × 10^−5^M concentration. The obtained solution was then stirred at RT for 3 h, after which time the excess HS-PEG was removed by ultracentrifugation. 10 mL plastic ultracentrifuge test tubes were used, at 13000 rpm for 25 min. After this time the product was found at the bottom of the rest tube a pellet of purple powder. The supernatant was carefully removed with a Pasteur pipette and the pellet redissolved in 10 mL bidistilled water. The process was repeated two more time. To obtain 10 × concentrated AgNP solution the pellet was redissolved in 1 mL bidistilled water Pegilation (surface coating with HS-PEGs) was obtained by treating at room temperature a given volume of the citrate-coated AgNP stock colloidal solution with a quantity of the chosen HS-PEG (added as a solid) so to reach a 2.0 × 10^−5^M concentration. The obtained solution was then stirred at RT for 3 h, after which time the excess HS-PEG was removed by ultracentrifugation. 10 mL plastic ultracentrifuce test tubes were used, at 13000 rpm for 25 min. After this time the product was found at the bottom of the rest tube a pellet of purple powder. The supernatant was carefully removed with a Pasteur pipette and the pellet redissolved in 10 mL bidistilled water. The process was repeated two more time. To obtain 10 × concentrated AgNP solution, at the end of the last cycle the pellet was redissolved in 1mL bidistilled water.

#### 3.3.4. Overcoating with Charged Polymers

The first step required the pegylation of AuNP with HS-PEGCOOH (mw 3000), that was carried out as described in the previous section. After the last ultracentrifugation cycle the AuNP@HS-PEGCOO(−) pellet was redissolved in 10 mL bidistilled water. More 10 mL portions were prepared from the same AuNP stock colloidal solutions, and these were gathered to prepare a 100 mL stock solution of the pegylated particles. After this, a 2 × 10^−3^ M solution of PAH in bidistilled water was prepared from the commercial product and the quantity needed to obtain a 2 × 10^−5^M solution was added with a micropipette to 50 mL of the AuNP@HS-PEGCOO(−) solution. The reaction mixture was alowed to equilibrate for 2 h at RT. After that time, two ultracentrifugation cycles with supernatant discard and redissolution in the same volume of bidistilled water were carried out to remove the PAH excess, obtaining a solution of AuNP@HS-PEGCOO(−)/PAH(+). The same procedure was carried out on the latter solution adding PSS at a 2 × 10^−5^M conc, thus preparing AuNP@HS-PEGCOO(−)/PAH(+)/PSS(−). For all ink preparations, a 10 mL volume of a solution of AuNP with the chosen charged coating was ultracentrifuged again, and the pellet redissolved in 1 mL bidistilled water.

#### 3.3.5. Ink Preparation

Inks were prepared by in 1.0 mL volumes by adding 200 μL ethylene glycol and 100 μL 2-propanol to 700 μL of 10 × concentrated AuNP solution, followed by gentle mixing on a reciprocal shaker at RT for 2 min. Larger volumes were prepared maintaining the same volume proportions.

#### 3.3.6. Inks with Ethyl Cellulose.

Due to the negligible solubility of EC in water, a different formulation was used. First, 5 mL of AuNP@HS-PEG_5000_ colloidal solution were added to 5 mL ethanol in ultracentrifuge tubes, followed by 13000 rpm 30 min ultracentrifugation, after which the supernatant was discarded and the pellet redissolved in 10 mL ethanol. A second ultracentrifugation followed, after which the pellet was redissolved in 0.5 mL ethanol to obtain a 10 × AuNP@HS-PEG_5000_ solution. This process was carried out simultaneously on 10 ultracentrifuge tubes and the 10 × portions were gathered to obtain 5 mL of concentrated ethanol solution. 1 mL ink samples were prepared by adding EC as a solid with weight/volume percent of 0.05–0.3% (0.5 to 3 mg/mL).

#### 3.3.7. Printing on Glass Slides

Forty μL of the chosen ink were dropcasted on a glass coverslide that was treated as described in Section 3.3.1. The coverslides still bearing a liquid drop of ink were transferred in an oven thermostatted at 40 °C carefully maintaining a horizontal set up. After 14 h the samples were considered dry and ready for characterization. A circular print of diameter 11–12 mm diameter was obtained (corresponding to a ~ 1cm^2^ area; in the rare event of prints with different shape or dimensions, these were discarded). When using the less viscous ethanol inks (EC containing inks), we drop the 40 μL drop inside a polydimethylsiloxane (PDMS) square well (1 cm side) drawn on the glass coverslide. This was done to limit the liquid spreading and maintain the print on the desired surface area (~ 1 cm^2^), when using such inks. The square well was drawn by hand with a thin brush and a freshly prepared PDMS solution, obtained by mixing in 10:1 ratio a SYLGARDTM 184 Silicone Elastomer base and its dedicated SYLGARD curing agent (both purchased from Sigma Aldrich, Milano, Italy). PDMS polymerisation was completed with 3 min at 140 °C.

#### 3.3.8. Determination of the Gold Content in Inks

The actual gold content in inks was determined to be sure that it was in the 0.16–0.21 mg/mL range. When lower concentrations were found, the ink was discarded and the colloidal aqueous solution used for its preparation was reconcentrated by ultracentrifugation. For quantitative analysis 200 μL of ink was treated with 500 μL aqua regia, observing immediated decoloration (Au oxidation). After 1 h, the sample was diluted to 10 mL with bidistilled water and then analysed for the gold content with an ICP-OES instrument.

#### 3.3.9. Sample Preparation for Scanning Electon Microscope (SEM) Imaging.

Printed glass coverslided were cut to obtain a 1 cm^2^ section, then coated with a 5 nm graphite layer by sputtering before imaging.

#### 3.3.10. Thermogravimetric Analysis (TGA)

One hundred mL of 1 × colloidal solution of AuNP with the chosen coating were subdivided into 10 ultracentrifuge tubes of 10 mL. After the UC, the pellets were redissolved in 1 mL bidistilled water, transferred to 2.5 mL Eppendorf vials and ultracentrifuged for 25 min at 13000 rpm. After discarding most of the supernatant, the 10 samples were carefully gathered using a micropipette in a single Eppendorf vial and dry blown with a nitrogen flow, obtaining 2–3 mg solid samples. Thermogravimetric analysis was performed in a Q5000 system by TA Instruments by heating the obtained powders in a Pt crucible from room temperature up to 600 °C at 5 °C/min under nitrogen flux. The data were elaborated by Universal Analysis v.4.5A by TA Instruments and the mass loss and temperature at mass loss values were evaluated considering the DTG signals

#### 3.3.11. Measurement of the Photothermal Effect

We used a FLIR E40 thermocamera with Flir Tools+ dedicated software for data acquisition and analysis. Thermal images were 320 × 240 pixels. For each thermogram a ROI was defined, that included the laser-irradiated area. Data analysis allowed to determine the maximum temperature inside the ROI (± 0.1 °C accuracy) for each thermal image. In a typical thermogram a thermal image was aquired every 0.25 s for 60–120s. All the laser sources have a beam waist of 1 cm, power was 90 mW for the 488 and 520 nm sources and 116 mW for the 720 nm source (ΔT was consequently normalized).

## 4. Conclusions

In this research work we have examined different polymeric coatings for spherical AuNPs, with the aim of maintaining their spectral stability both in liquid inks and in dry prints, as this is a mandatory feature for their use in secure writing of photothermally readable information. The traditional linear HS-PEG coatings (mw 2000–20000) were found unsuitable, as they demonstrated uncapable to avoid agglomeration of the coated AuNP both in the liquid ink and in the dry prints. Moreover, we also found a counter-intuitive trend of agglomeration promotion with the increase of the polymer length (i.e., mw), that is due to a less efficient grafting of HS-PEG on AuNP surface with increasing mw. Also the tested dispersant polymer, EC, revealed unefficient in keeping AuNP separated in the prints. On the contrary, the presence of a poorly hydrophilic matrix as EC promotes segregation of the hydrophilic AuNP (coated with HS-PEG_5000_) and their agglomeration. Effective coatings for maintaining a sufficent interparticle separation and avoid LSPR hybridization were instead found when AuNP bearing a first HS-PEGCOO(-) coating (necessary to introduce an high negative ζ-potential) were overcoated with the positively charged ionic polymer PAH or with PAH and a further overcoating with the negatively charged ionic polymer PSS. In both cases the high ζ-potential and the thickened coating layers contributed to keep AuNP at a sufficiently large distance to avoid the hybridization of their LSPR bands. Accordingly, prints with AuNP@HS-PEGCOO(−) have a poorly efficient protection against AuNP agglomeration and give a photothermal information reading radically different with respect to what expected from their absorbance as inks; morevoer, their photothermal information reading changes with time. Affordable and time-stable prints are instead obtained both with AuNP@HS-PEGCOO(−)/PAH(+) and AuNP@HS-PEGCOO(−)/PAH(+)/PSS(−), from which durable multiwavelength photothermal barcodes can be read after the definition of a number of security keys.

## Figures and Tables

**Table 1:** Dimensional and chemical-physical data of PEG-coated AuNP.

	Citrate^a^	HS-PEG_2000_	HS-PEG_5000_	HS-PEG_10000_	HS-PEG_20000_
r_hyd_ (nm)^b^	16(1)	23(1)	54	74	93
ζ (mv)	−32(2)	−13(1)	−5(1)	−8(1)	−1(1)
%w/w^c^		10.69(5)	21.95(6)	21.72(3)	29.41(7)
n/NP^d^		1805	1758	890	651

^a^ Parent AuNP, with no PEG coating ^b^ hydrodynamic radius determined in water. ^c^ % of PEG weight in dry samples; water is adsorbed on the solid samples used for TGA measurements, the % Au is calculated by subtracting the % of PEG and % of water to 100 (see SI for TGA profiles and details); ^d^ number of polymer chains per NP.

**Table 2:** Dimensional and chemical-physical data of AuNP coated with charged polymers.

	AuNP@HS-PEGCOO(−)	AuNP@HS-PEGCOO(−)/PAH(+)	AuNP@HS-PEGCOO(−)/PAH(+)/PSS(−)
r_hyd_(nm)^a^	58(2)	178(8)	197(6)
ζ (mv)	−22(2)	+36(2)	−27(3)
%w/w^b^	12.27(4) Au: 86.61(4)	18.17(3) Au: 76.73(3)	20.27(4) Au: 74.46(4)
Au conc^c^	1.07 × 10^−3^ M (0.211 mg/mL)	7.88 × 10^− 4^M (0.155 mg/mL)	9.38 × 10^−4^M (0.185 mg/mL)
λ_max_(nm)^d^	524	534	526

^a^ Hydrodynamic radius determined in water. ^b^ % of organic material and % of gold in the dry products, determined by TGA; water is adsorbed on the solid samples, the % Au is calculated by subtracting the % of organic matter and % of water to 100; ^c^ Au concentration (by ICP-OES) in the 10-fold preconcentrated aqueous solutions, before ink formation; ^d^ maximum of absorption measured in the ink solvent mixture.

## Supplementary Materials

The following are available online at https://www.mdpi.com/1420-3049/25/11/2499/s1, SM1. Inks ageing for AuNP@HS-PEG_X000_ (X = 5, 10, 20), spectra and photographies; SM2. TGA (thermogravimentric analysis) profiles; SM3. Absorption spectra of prints with AuNP@HS-PEGmw; SM4. Larger TEM image of AuNP@HS-PEG_5000_ redissolved in water after printing; SM5. Absorption spectrum of AuNP@HS-PEG_5000_ in ethanol; SM6. Absorption spectra of AuNP@HS-PEG_5000_ in EtOH with added EthylCellulose; SM7. SEM image of a 14 days aged print with AuNP@HS-PEGCOO(−)/PAH(+) ink; SM8. UV-Vis absorbance spectra and TEM images of redissolved AuNP@HS-PEGCOO(−) prints; SM9-Thermograms
