# Supplementary material for: Suitable Polymeric Coatings to Avoid Localized Surface Plasmon Resonance Hybridization in Printed Patterns of Photothermally Responsive Gold Nanoinks
Source: Molecules. 2020 May 27;25(11):2499. doi: 10.3390/molecules25112499 (PMC7321298; doi:10.3390/molecules25112499)
Supplement: Supplementary file 1 [file molecules-25-02499-s001.pdf]

Supplementary Materials

Suitable Polymeric Coatings to Avoid Localized Surface Plasmon Resonance Hybridization in Printed Patterns of Photothermally Responsive Gold Nanoinks

Piersandro Pallavicini [1,*] Lorenzo De Vita [1] Francesca Merlin [1] Chiara Milanese [1] Mykola Borzenkov [2] Angelo Taglietti [1] and Giuseppe Chirico [2]

[1] Dipartimento di Chimica, Università di Pavia, viale Taramelli, 12–27100 Pavia, Italy; lorenzodevita01@universitadipavia.it (L.D.V.); francesca.merlin01@universitadipavia.it (F.M.); chiara.milanese@unipv.it (C.M.); angelo.taglietti@unipv.it (A.T.)

[2] Dipartimento di Fisica "G.Occhialini", Università Milano Bicocca, p.zza della Scienza, 3–20126 Milano, Italy; mykola.borzenkov@unimib.it (M.B.); giuseppe.chirico@mib.infn.it (G.C.)

* Correspondence: piersandro.pallavicini@unipv.it; Tel.: +39-0382-987-336

SM1. Inks ageing for AuNP@HS-PEGX000 (X = 5, 10, 20)

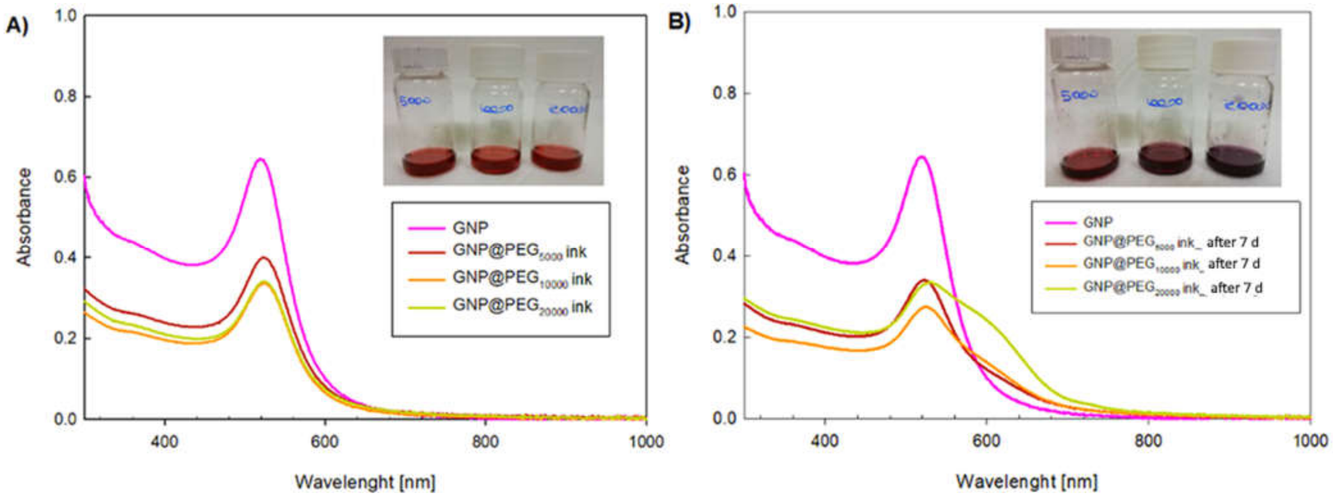

A: Absorption spectra of GNP in water (pink spectrum) and absorption spectra of inks 1 d after preparation; inset: photography of the three inks just before recording the spectra

B: same, after 7 days

SM 2. TGA (thermogravimentric analysis) for AuNP@HS-PEG$_{X000}$ (X = 2, 5, 10, 20)

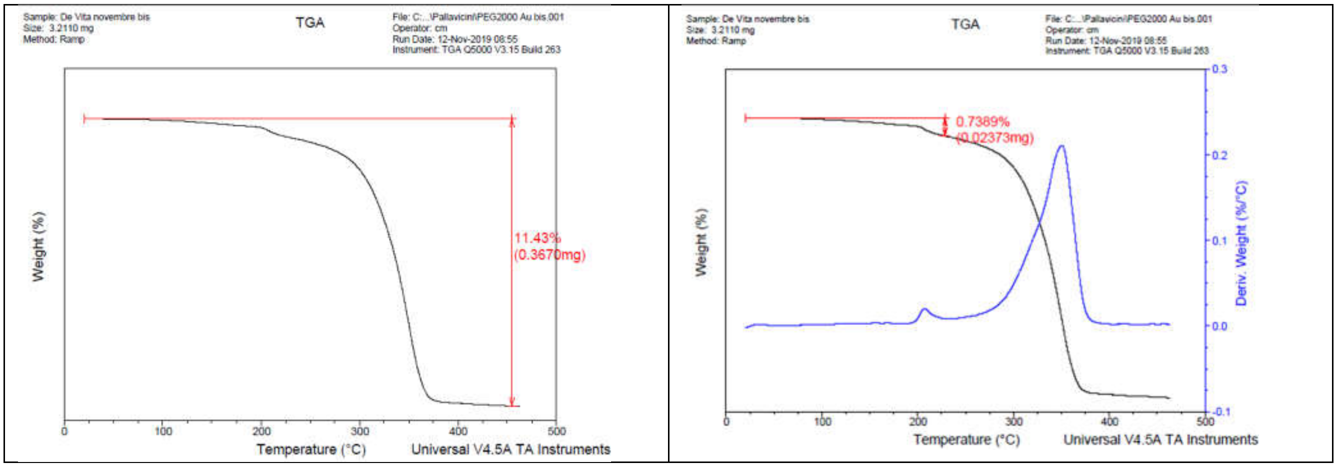

SM2A: left, TGA on AuNP@HS-PEG$_{2000}$; right, determination of the solvent (water) content.

Au content in a 100 g sample of AuNP@HS-PEG$_{2000}$: 100-11.43 = 88.57g
HS-PEG$_{2000}$ content in a 100 g sample of AuNP@HS-PEG$_{2000}$: 11.43-0.74 = 10.69g (5.345×10^{-3} mol)
mass of a spherical AuNP of 17 nm diameter: 4.97×10^{-17}g → number of AuNP in a 100 g sample of AuNP@HS-PEG$_{2000}$ = 1.782×10^{18} → mol of AuNP = 2.959×10^{-6} → number of HSPEG$_{2000}$ per AuNP = 5.345×10^{-3} mol/2.959×10^{-6} = 1805

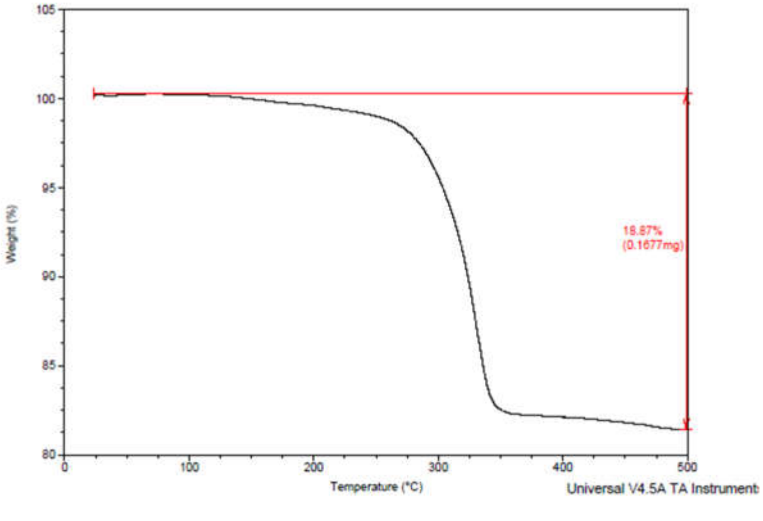

SM2B: TGA on AuNP@HS-PEG$_{5000}$

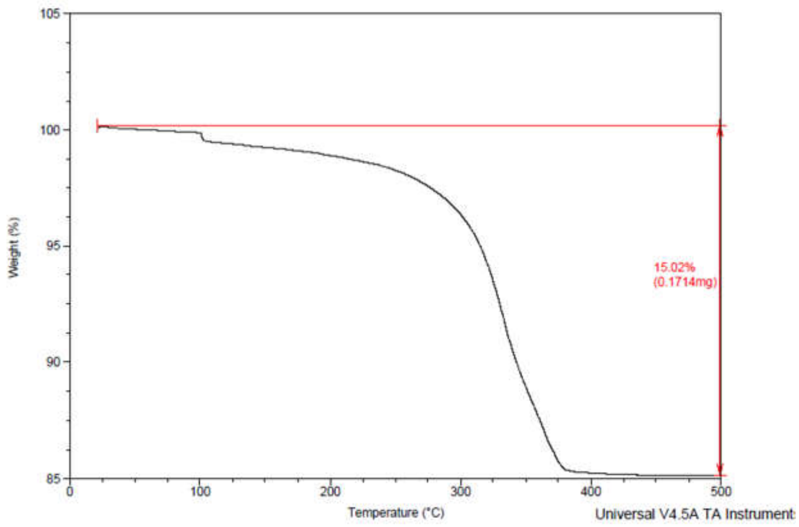

SM2C: TGA on AuNP@HS-PEG10000

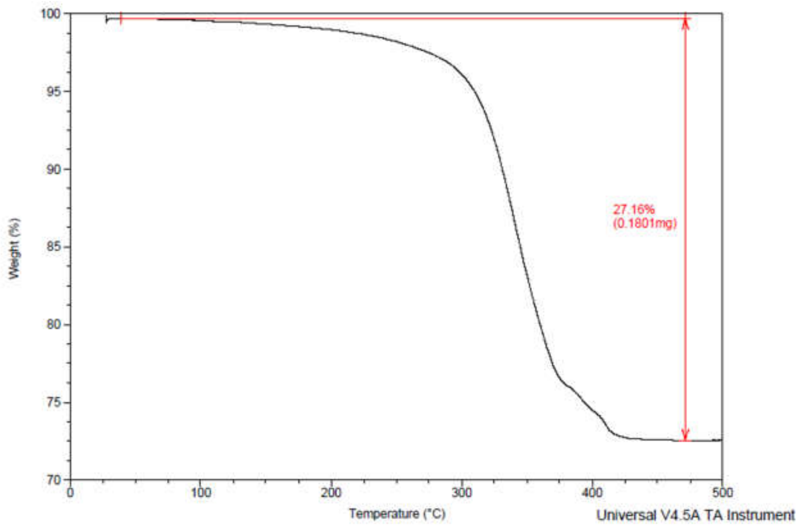

SM2D: TGA on AuNP@HS-PEG20000

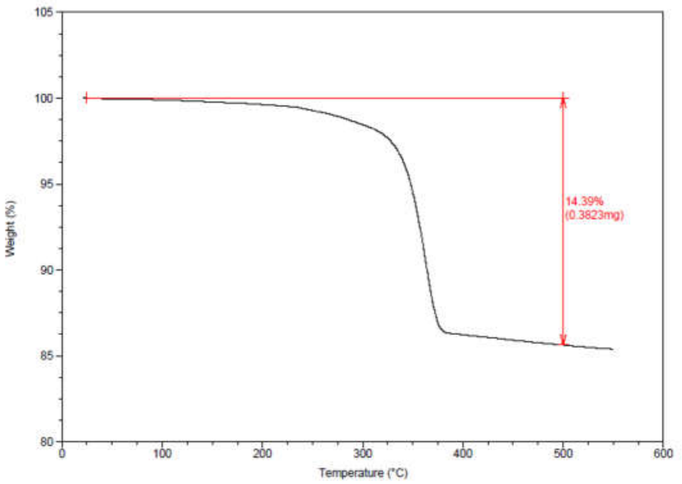

SM2E. TGA on AuNP@HS-PEGCOO(-)

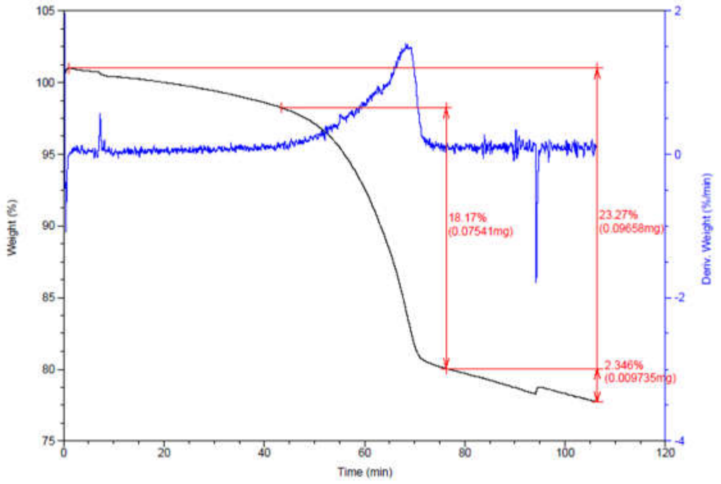

SM2F. TGA on AuNP@HS-PEGCOO(-)/PAH(+)

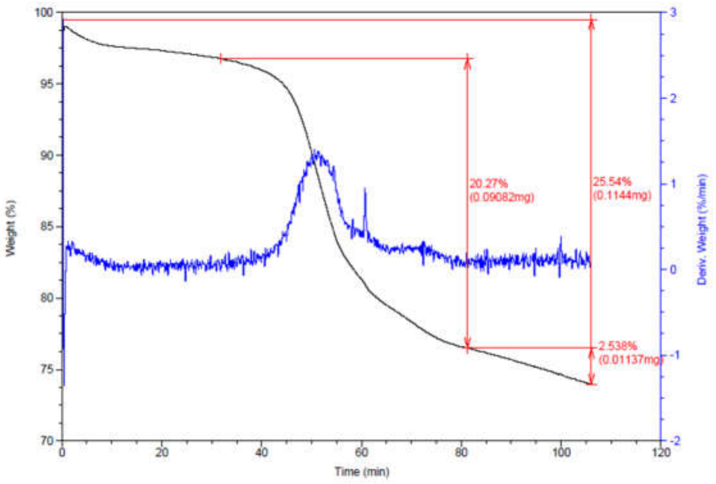

SM2G. TGA on AuNP@HS-PEGCOO(-)/PAH(+)/PSS(-)

SM3 – prints with AuNP@HS-PEGs

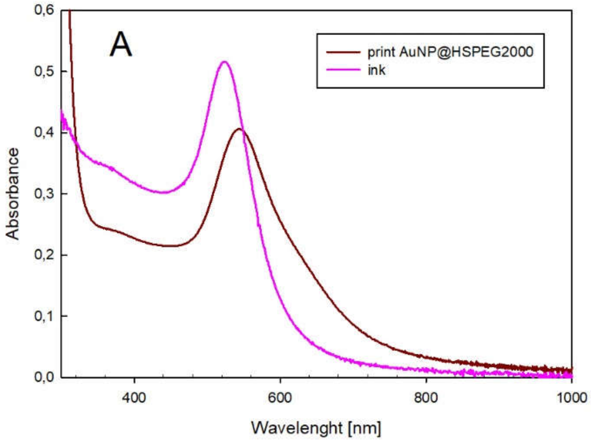

SM3A: Absorption spectra of AuNP@HS-PEG$_{2000}$ ink (pink) and a print on glass after 1 day

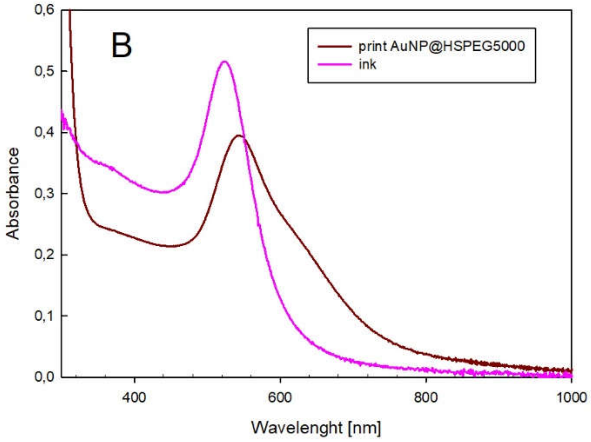

SM3B: Absorption spectra of AuNP@HS-PEG$_{5000}$ ink (pink) and a print on glass after 1 day

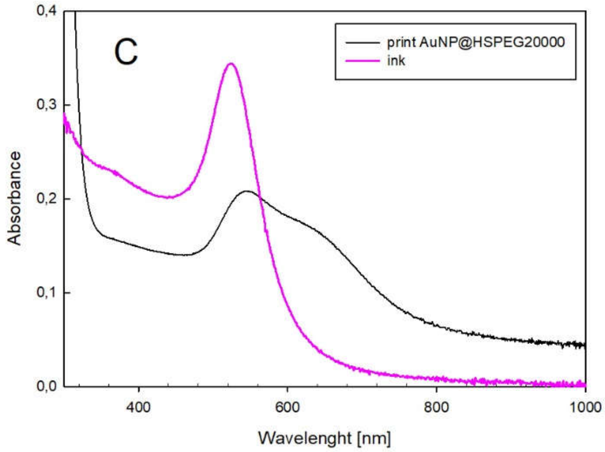

SM3C: Absorption spectra of AuNP@HS-PEG$_{10000}$ ink (pink) and a print on glass after 1 day

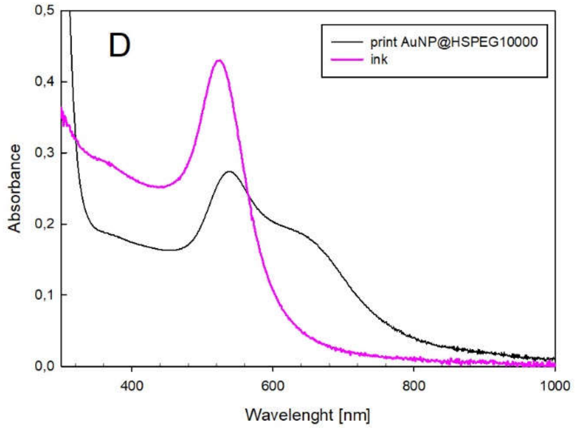

SM3D: Absorption spectra of AuNP@HS-PEG$_{20000}$ ink (pink) and a print on glass after 1 day

SM4. Larger TEM image of AuNP@HS-PEG5000 redissolved in water after printing

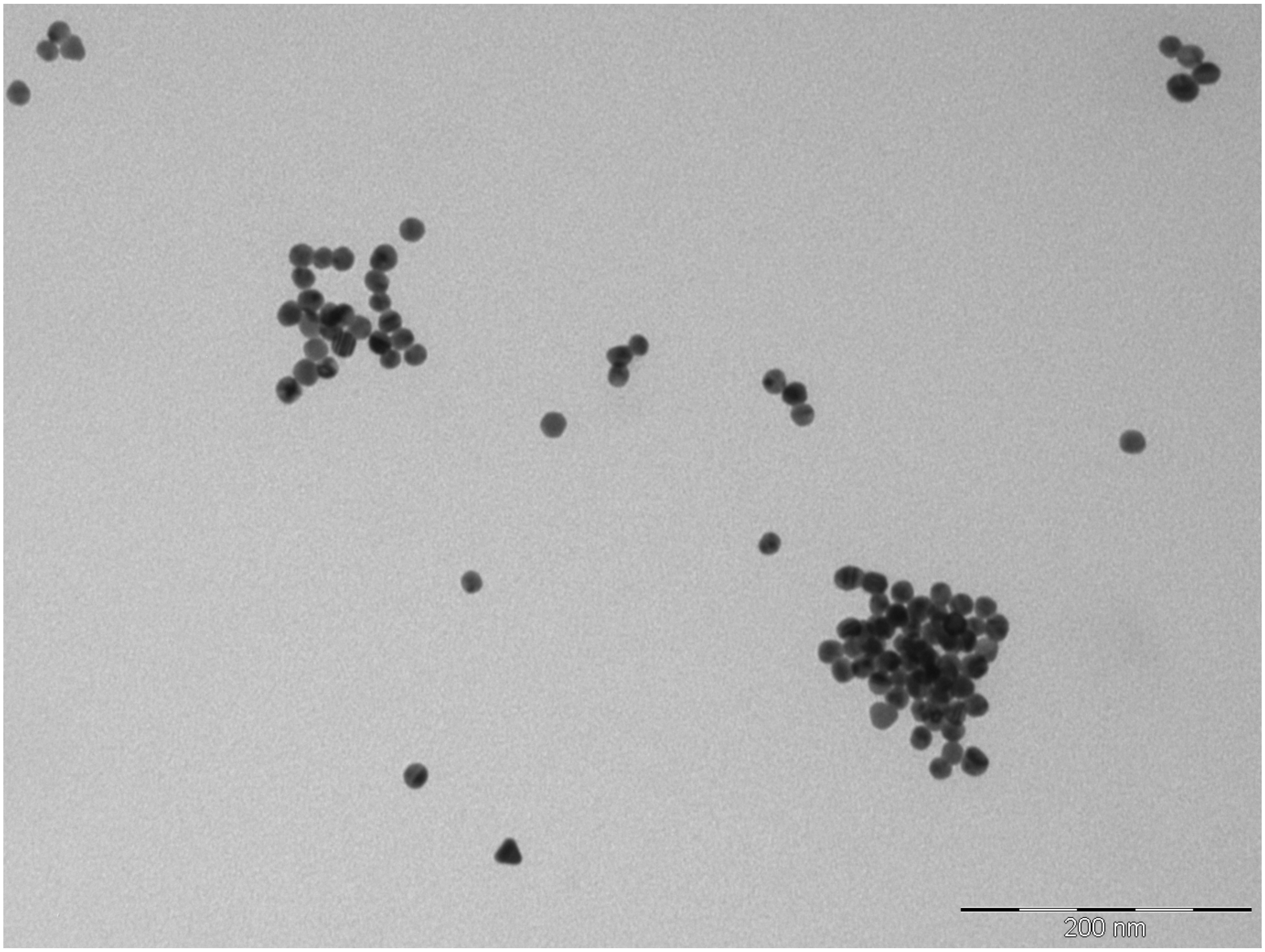

This image is the same as Figure 4G, main text, with larger dimensions

SM5. Absorption spectrum of AuNP@HS-PEG$_{5000}$ in ethanol

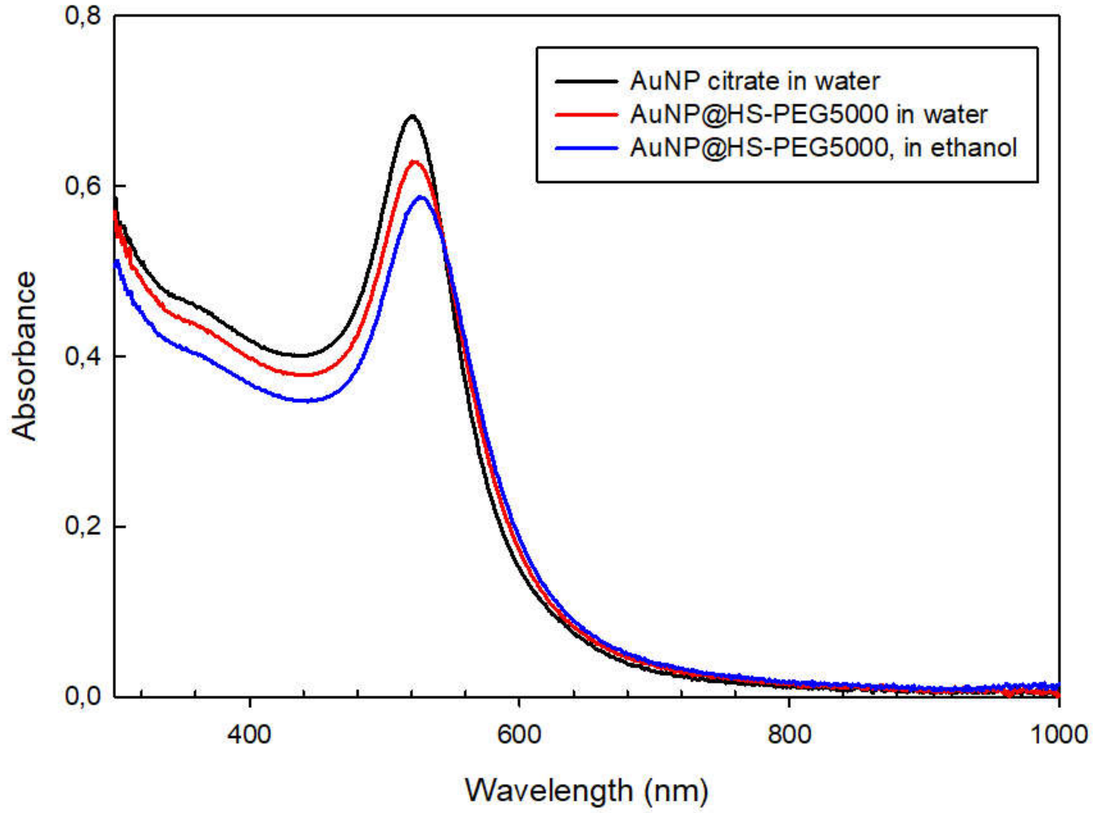

The spectrum of AuNP@HS-PEG$_{5000}$ in ethanol (blue) was recorded after 2 h from preparation. It did not change after a further 22 h (spectrum not reported – even after 4 d, no significant changes were observed, see pink spectrum in Figure SM6B). Spectra of citrate-coated AuNP in water (black) and of AuNP@HS-PEG$_{5000}$ in water (red) are also included for comparison

SM6. AuNP@HS-PEG5000 in EtOH with added Ethyl Cellulose

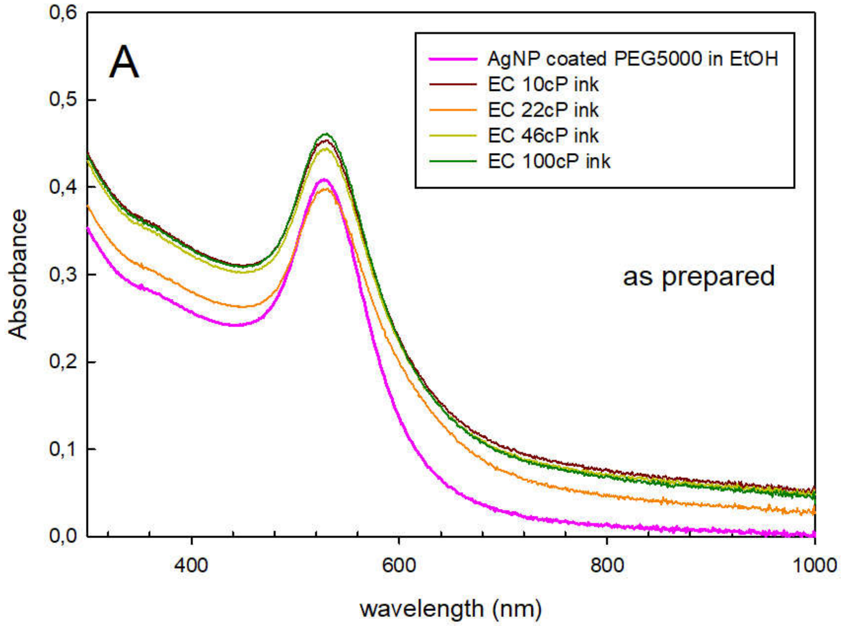

A: as prepared AgNP@HS-PEG_{5000} in ethanol (pink) and in solutions (0.1%) with EC of different viscosity

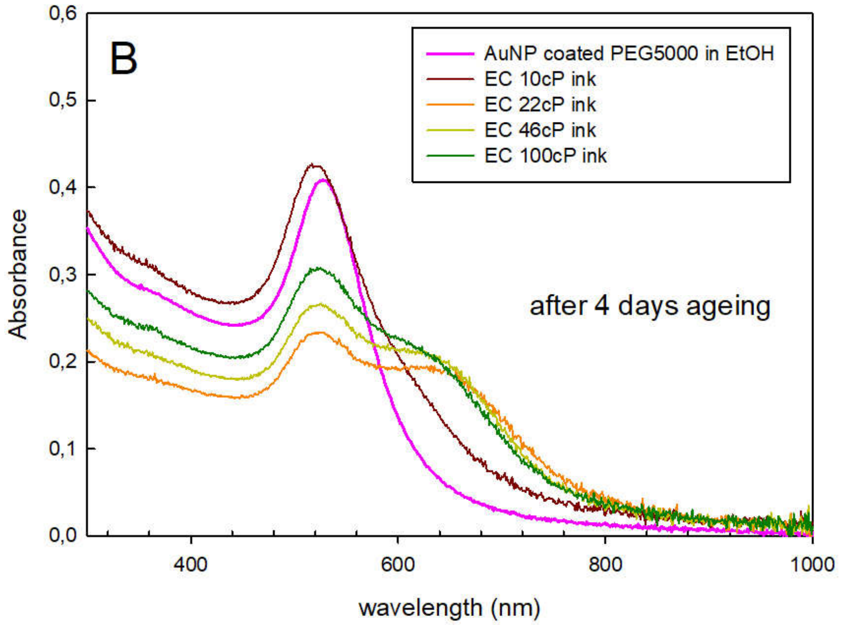

B: same solutions as in A, after 4 days

SM7. SEM image of a 14 d aged print with AuNP@HS-PEGCOO(-)/PAH(+) ink

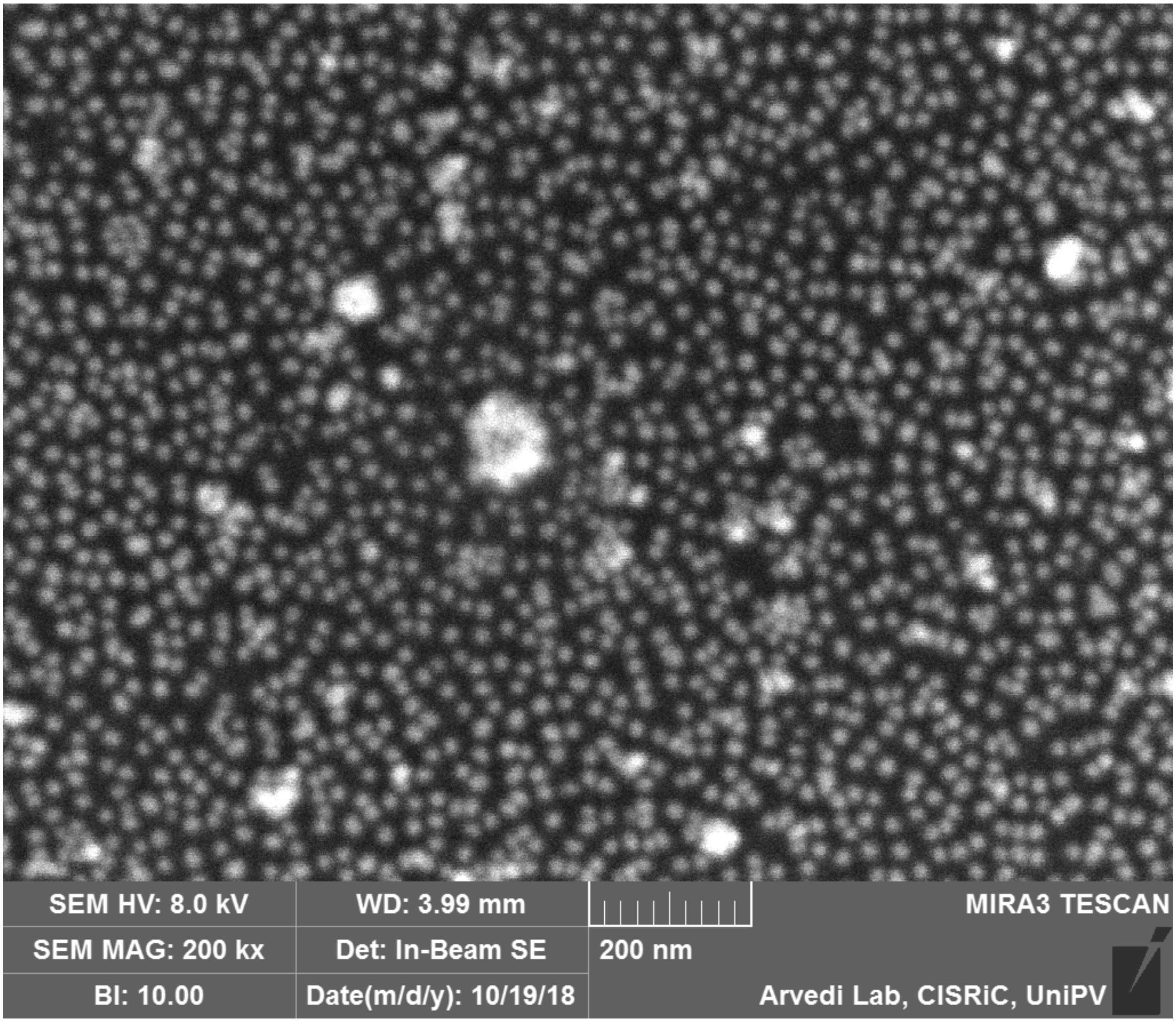

SM8 – UV-Vis absorbance spectra and TEM images of redissolved AuNP@HS-PEGCOO(-) prints

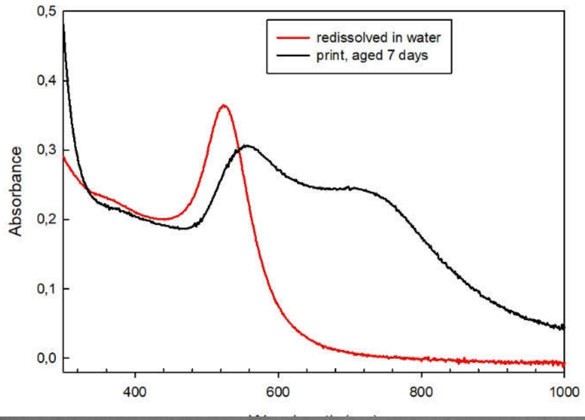

Absorption spectrum of a print with AuNP@HS-PEGCOO(-) ink (7 days aged) and of the same print redissolved in water.

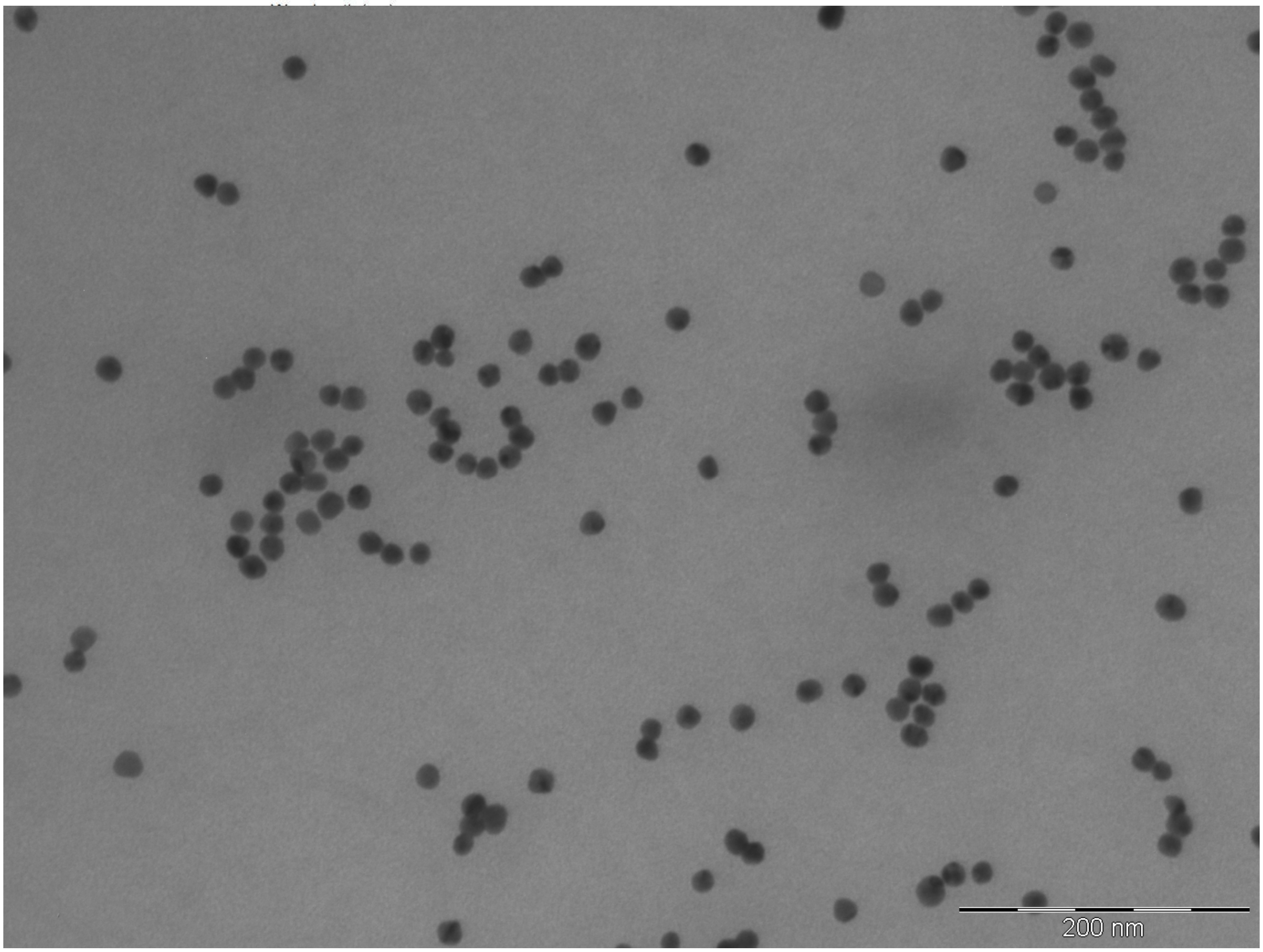

SM8B – TEM image obtained from the redissolved ink
